# Understanding Maize Response to Nitrogen Limitation in Different Light Conditions for the Improvement of Photosynthesis

**DOI:** 10.3390/plants10091932

**Published:** 2021-09-16

**Authors:** Aleksandra Urban, Paweł Rogowski, Wioleta Wasilewska-Dębowska, Elżbieta Romanowska

**Affiliations:** Department of Molecular Plant Physiology, Institute of Environmental Biology, Faculty of Biology, University of Warsaw, Miecznikowa 1, 02096 Warsaw, Poland; aleksandra.urban@student.uw.edu.pl (A.U.); progowski@biol.uw.edu.pl (P.R.); wiolaw@biol.uw.edu.pl (W.W.-D.)

**Keywords:** photosynthesis, nitrogen and light intensity, bundle sheath and mesophyll chloroplasts of C4 plant maize, enzymes and regulation

## Abstract

The photosynthetic capacity of leaves is determined by their content of nitrogen (N). Nitrogen involved in photosynthesis is divided between soluble proteins and thylakoid membrane proteins. In C4 plants, the photosynthetic apparatus is partitioned between two cell types: mesophyll cells and bundle sheath. The enzymes involved in the C4 carbon cycle and assimilation of nitrogen are localized in a cell-specific manner. Although intracellular distribution of enzymes of N and carbon assimilation is variable, little is known about the physiological consequences of this distribution caused by light changes. Light intensity and nitrogen concentration influence content of nitrates in leaves and can induce activity of the main enzymes involved in N metabolism, and changes that reduce the photosynthesis rate also reduce photosynthetic N use efficiency. In this review, we wish to highlight and discuss how/whether light intensity can improve photosynthesis in maize during nitrogen limitation. We described the general regulation of changes in the main photosynthetic and nitrogen metabolism enzymes, their quantity and localization, thylakoid protein abundance, intracellular transport of organic acids as well as specific features connected with C4 photosynthesis, and addressed the major open questions related to N metabolism and effects of light on photosynthesis in C4 plants.

## 1. Introduction

**Figure 1:**
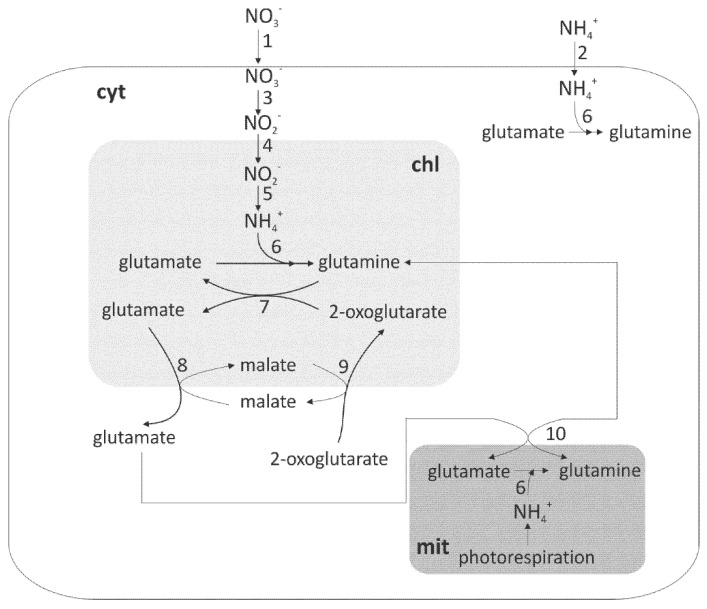
Schematic representation of plant nitrate assimilation. Reactions take place in mitochondrium (mit), chloroplast (chl), and cytoplasm (cyt). Enzymes and transporters are symbolized by numbers: 1—nitrate transporter, 2—ammonium transporter, 3—nitrate reductase, 4—nitrite transporter, 5—nitrite reductase, 6—glutamine synthetase, 7—glutamate synthase, 8—plastidic glutamate–malate translocator, 9—plastidic 2-oxoglutarate–malate translocator, and 10—mitochondrial glutamate–glutamine translocator. The major pathway of nitrate assimilation is shown in bold. Modified from [10].

Nitrogen is the most expensive nutrient supplied to growing plants. Therefore, better nitrogen-use efficiency (NUE) is one of the main goals of current research on plant nutrition [1]. The chemical environment, soil type, and species may change the ability to capture nitrogen from the soil. Such factors as precipitation, temperature, soil type, and pH have influence on nitrogen availability. Thus, the preferred form of nitrogen for uptake depends on the adaptation of a plant to soil conditions [2]. The N forms taken up by plants are inorganic ones, such as nitrate or ammonium (Figure 1). Maize (*Zea mays* L.) is a C4 plant of NADP-ME (nicotinamide adenine dinucleotide phosphate malic enzyme) subtype. It is one of the most exploited crops in the world and ranked as the third major cereal crop after wheat and rice. The high yield of maize largely contributed to the global food production and the use of this crop for producing bioenergy [3]. C4 metabolism allows for efficient photosynthesis and subsequently green biomass and crop yield, especially under conditions of maximal nitrogen supply [4]. Mineral nitrogen nutrition is one of the most significant factors that influence the productivity and characteristics of plants [5]. Optimal nitrogen nutrition is essential for the development of the root system and overground biomass. Hence, the dynamics of N uptake and N partitioning in the plants were of great interest among researchers and it is considered important to identify the critical steps controlling the NUE of plants. Numerous studies confirmed that nitrogen mineral fertilizers increase the yield of maize grains [6,7]. Crops often take up only less than half the amount of N applied, while the rest is lost as gaseous nitrous oxide emissions and nitrate leachate or added to soil organic matter, which reduces NUE in agricultural systems [8]. The large demand of plants for inorganic nitrogen fertilizers often results in high application rates during growth. Therefore, an understanding of N nutrition is of fundamental importance in basic and applied plant sciences. Plants use light energy to convert atmospheric CO_2_ into essential organic compounds required for their growth and maintenance. Accordingly, the production of plant biomass by terrestrial vegetation was observed to be strongly and positively correlated with the canopy absorption of photosynthetically active radiation, which is known as light-use efficiency. Hence, a strong positive correlation was found between the light-saturated rate of photosynthesis (Pn) and leaf nitrogen content [9].

Changes in a specific leaf area (SLA; projected leaf area per unit leaf dry mass) and nitrogen partitioning between proteins within leaves occur during the acclimation of plants to their growth irradiance. Plants can acclimate to the light environment [11] in the following ways: (1) by changing the fraction of biomass invested in leaves, stems, and roots; (2) by modulating the leaf area per unit of biomass invested in leaves, by altering their anatomy; or (3) by changing the relative investment of nitrogen between photosynthetic components. Plants that are grown under high light (HL) intensity generally have thick leaves with a low SLA [12]. This results in an increase in the number of chloroplasts and the amount of photosynthetic enzymes and enhances the photosynthetic capacity of leaves per unit area. However, due to the presence of more biomass in a given area, the increase in photosynthetic capacity of HL leaves may be associated with less light capture per unit biomass at lower irradiances. The most important features of leaves grown under HL intensity in comparison with leaves grown under low light (LL) are the following: (1) less chlorophyll per unit of nitrogen; (2) a higher chlorophyll a/b ratio; (3) an increased electron transport capacity per unit of chlorophyll; (4) a slightly greater ratio of electron transport capacity to Rubisco activity [13]. In a previous study [14], we observed that maize grown under LL and HL intensity had a similar rate of photosynthesis in LL conditions, whereas the rate of photosynthesis in HL conditions was relatively higher and independent of the light conditions during growth. The nutrient supply was found to be adequate for plant growth. Such plasticity in photosynthetic light acclimation may be advantageous when LL-grown plants are exposed to HL intensity and reach the level of photosynthesis observed in HL-grown plants. It was of particular importance in maize to associate the differences in the rate of photosynthesis with a better nutrition apply. When N deficiency occurs, maize photosynthesis is reduced and consequently has a profound influence on grain yield [15]. Reduced N is also associated with lower protein content in the seeds and vegetative parts [16]. Nitrogen content in the seeds further determines the germination efficiency and survival of young seedlings. A low amount of soil and light may inhibit the growth of maize in the early developmental stage, whereas optimal nutrition in this stage has a positive effect on growth and yield [17].

The grain yield of maize greatly varies depending on the dynamics of initial growth, which is expressed as the accumulation of dry matter (DM) in the five- to six-leaf stage [6]. Exposure to stress in the early developmental stage is the primary reason for the reduction of maize yield. Additionally, light deficiency during seedling development inhibits the growth due to reduced uptake of water and nutrients, particularly nitrogen. In earlier studies, authors [18] reported that maize is highly sensitive to nutrient deficiencies, especially at the early growth stages. Thus, ensuring optimal nutrition during the early vegetative period may improve the yield. This was also proven by [7] who found in a greenhouse experiment that inadequate nitrogen nutrition caused a reduction in seed yield. In the field experiments, the combination effects of N fertilizer and planting density on maize yield, NUE, and the characteristics of canopy light absorption and radiation use efficiency (RUE) show that increased planting density with reduced basal N application might be beneficial for maize high yield [19]. Crop scientists are trying to correlate yield to how plants intercept light and allocate dry matter to grains. It was found that canopy foliar nitrogen content is a determinant of RUE. Uniform foliar N distribution increased with the canopy leaf area index (LAI) and with the deficiency of foliar N [20]. Maize radiation use-efficiency response to optimally distributed foliar-nitrogen-content depends on the canopy leaf-area index. The effect of plant densities was not evident in the early stages of growth but became prominent in the plants with different heights [21]. As density increased, it accelerated leaf senescence, reducing photosynthesis of the plants due to a fall in LAI. Though, there is no difference in LAI between plants of different heights grown in varying densities, the shorter plants maintain their LAI in later life stages compared to medium and tall maize varieties. Similarly, the low maize variety had the highest chlorophyll content at all densities compared to the medium and tall maize cultivars.

The optimum rate and time of N application enhanced yield productivity and NUE by reducing the environmental influence. The application of nitrogen beyond the optimal requirement could not increase the yield of maize, but may led to an elevated level of NO_3_^−^ in the soil and increased the susceptibility of plants to NO_3_^−^ loss by leaching. It was shown that 50% or even more of the applied N remains unavailable to a crop due to N loss through leaching [22]. If the N supply exceeds the plant requirements, it will result in loss of NH_3_. Compared with farmers’ practices, application of N following 10–15 days of planting can significantly increase yield. NUE even under low levels of nitrogen at two to three applications considerably enhances N absorption. Application of nitrogenous fertilizer may increase plant yield, probably by developing leaf area and thereby increasing the physiological growth indices [23]. The type of nitrogen fertilizer, its dose, and light intensity may determine the dynamics of early growth in maize, which is expressed by the growth rate for DM production and different physiological parameters influencing the rate of photosynthesis [24]. Increased N nutrition usually causes some anatomical changes in leaves. The association between the effects of N nutrition on the proportions of proteins and the reflection of these effects in leaf anatomy need to be evaluated under various environmental conditions. Several studies have reported that changes in chloroplast ultrastructure were observed when leaves were transferred from low-irradiance to high-irradiance conditions [25]. It is unknown whether photon flux density (PFD) can cause nonuniform distribution of leaf nitrogen and induce changes in the anatomy of leaves. Light intensity and nitrogen concentration influence the content of nitrate in cultivated leaf vegetables. An increase in light intensity can induce the activity of the main enzymes involved in nitrate metabolism [26]. Nitrogen concentration in fertilizers may also influence the accumulation of other nutrients by plants, which influences their growth because those nutrients are needed for development and proper functioning, ref. [27] and elements such as P, K, Mg, Mn, Mo, etc., often tied to the N cycle, may be critical for the growth rate.

The major functional parameters reflect the adaptation of plants to light and N availability during growth and the increase in understanding of leaf economics (C–N stoichiometry) and plant growth strategies. The strategies would be related to higher C:N ratios in the plant material, particularly for the development of specific energy crop lines, where C content is more significant than protein content [28]. Stimulation of uptake and assimilation of nitrogen by photosynthesis [29] ensures that N uptake is correlated with C status.

The rates of photosynthesis and N concentration increase when plants are moved from a shaded location to sun-exposed locations [30]. Light is important for the partitioning of N in photosynthetic complexes [31] and, hence, light absorption influences the photosynthetic electron transport chain and enhances photosynthetic productivity [32]. Therefore, the availability of both nutrients and light affects the activity of photosynthetic enzymes [33]. High irradiance and nitrogen content are needed for a plant to reach the maximum rate of photosynthesis per unit of nitrogen. The effects of nitrogen nutrition and PFD on the organization of the photosynthetic apparatus in maize leaves were investigated [34], but the processes predicting the photosynthetic capacity of C4 plants are not studied so far. Variations in net photosynthesis based on growth conditions are related to concurrent changes in leaf nitrogen content (N) and allocation of nitrogen between metabolic and structural protein pools. As leaf structures are highly dependent upon the biomass investment in DM, structural leaf N is expressed per unit of DM (gN g^−1^ DM). Metabolic leaf N associated with leaf photosynthesis is expressed per unit area (gN m^−2^), since both light capture and CO_2_ exchange are area-based phenomena. Structural leaf N pool is found in cell walls, nucleic acids, other non-photosynthetic nitrogenous components as cytosolic proteins and amino acids, ribosomes, mitochondria, and so on. It was shown that a greater fraction of leaf mass in cell walls is typically associated with a lower fraction of leaf nitrogen (N) invested in photosynthetic proteins and lower within-leaf CO_2_ diffusion rates, due to the thicker cell walls of mesophyll cells, which in turn reduces the efficiency of photosynthesis [9]. Generally, higher nitrogen content is associated with higher rates of photosynthesis, which is attributed to the large amount of leaf organic nitrogen (up to 75%) present in the chloroplasts [34].

Despite the strong association between photosynthesis and nitrogen, the ratio of the photosynthesis rate and amount of (organic) nitrogen in the leaf, photosynthetic nitrogen-use efficiency (PNUE), and ratio of CO_2_ assimilation rate and leaf organic nitrogen content do not remain constant in plants. Different molecular and physiological factors cause variations in PNUE [35], and as a result, large differences are observed between plant species. Accordingly, C4 plants are characterized by a greater rate of photosynthesis than C3 plants with the same N concentration [36]. In addition, a higher NUE was found in the C4 pathway than in the C3 pathway [37]. While the biochemistry of C4 photosynthesis was largely solved in the middle of the last century, renewed interest in engineering C4 photosynthesis in crops has jump-started systematic analysis of C4 photosynthesis especially light regulation of plants metabolism.

## 2. Nitrogen, Light, and Photosynthesis in C3 and C4 Plants

Brown [38] hypothesized that C4 species utilize N more efficiently than C3 species, which was also confirmed by Makino et al. [39] for maize (C4), wheat (C3), and rice (C3) plants. C4 plants were also reported to show increased water- and light-use efficiency compared to C3 plants [4]. The main difference in the NUE of these plant species appears to be based on N partitioning among leaf proteins and the related carbon assimilation pathway [40]. C4 leaves change their photosynthetic characteristics in response to light conditions during growth [41,42]. However, the responses of C4 plants may differ depending on the decarboxylation subtype, plant type (monocots or dicots), and even the species. It was shown [43] for *Amaranthus cruentus* plants that grow under different light and nitrogen conditions changes the investment of N into the photosynthetic components. In low light (LL) leaves, the chlorophyll content per leaf area was greater and the chlorophyll a/b ratio was lower compared to high light (HL) leaves. This indicates that LL leaves invest more N into their light-harvesting systems. Allocation of nitrogen to Rubisco was significantly higher in HL and high-N leaves than in other leaves. On the other hand, N allocation to C4 enzymes phosphoenolpyruvate carboxylase (PEPC) and pyruvate orthophosphate dikinase (PPDK) was unaffected by growth conditions.

**Figure 2:**
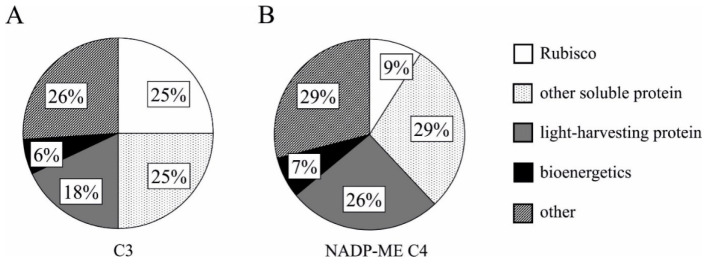
Nitrogen allocation on the percentage of different N components within the leaf in C3 (**A**) and NADP-ME C4 (**B**) plants. Modified by Mu and Chen [17].

In C3 species, the proportion of nitrogen allocated to Rubisco, a major leaf protein, increases with an increase in leaf protein levels, and the amount of this enzyme correlates strongly with photosynthesis rate which largely responds to the produced biomass. C4 plants invest more N into light harvesting protein (Figure 2) and have a higher light energy convention and electron transport rate, whereas they invest less to Rubisco, compared with C3 plants. 

This results in high photosynthetic efficiency of C4 plants [17]. Identifying the regulatory elements that control the balance between N allocation to maintain photosynthesis and the reallocation of the remobilized N to sink organs, such as young developing leaves and seeds, is of importance in C3 and C4 species, particularly when N becomes a limiting factor. The Kranz-type leaf anatomy typically associated with C4 photosynthesis comprises two photosynthetic cell types, bundle sheath (BS) and mesophyll (M) cells, which differ in their CO_2_ assimilation functions [44,45]. These two cell types provide structural and functional compartmentalization for separate sets of carboxylation and decarboxylation reactions essential for the C4 pathway. Rubisco (ribulose 1,5-*bis*-phosphate carboxylase) is specific to the BS cells of C4 leaves. This enzyme accumulates abundantly in the chloroplasts of C3 species and represents as much as 50% of the total soluble proteins [46] or 20% of leaf nitrogen [47]. In the leaves of most C4 plants, Rubisco accumulates only in BS cells surrounding the vascular system and is not present in mesophyll cells. Thus, its levels in C4 plants tend to be significantly lower compared to C3 plants (about 50–80%) [48]. The BS compartment has at least 15–20 times higher CO_2_ concentration than the surrounding atmosphere [49]. High photosynthetic capacity and, therefore, high biomass productivity of C4 plants as compared with C3 plants can be attributed to the CO_2_-concentrating mechanism (CCM) in BS chloroplasts. At temperatures of 20–30 °C, Rubisco in C4 plants encounters almost saturated CO_2_ concentrations, thus photorespiration is very low [50]. The C4 plants exhibit little evidence of photorespiratory CO_2_ production because: the CO_2_ compensation point of maize leaves was shown to be less than 10 ppm, apparent photosynthesis is relatively insensitive to changes in O_2_ concentration, and no post-illumination burst of CO_2_ can be detected from maize leaves [51,52]. However, the photorespiratory events occurring in mesophyll and bundle sheath cells are also dependent, similar to photosynthesis, on the movement of metabolites between the two cell types. Although photorespiration is low in the NADP-ME subtype of C4 plants, this process is very important for cell metabolism: it prevents ROS accumulation during stress; it can dissipate excess reducing equivalents and energy; and it can provide an internal CO_2_ pool [53,54]. Thus, C4 species require 25–30%, as much Rubisco as C3 species, to maintain a given CO_2_ assimilation rate. This reduced requirement explains the lower Rubisco content of C4 plants relative to C3 species of similar life forms [55]. The physiological significance of the biochemical diversification of C4 photosynthesis is not clearly understood. Foliar N allocation in C4 leaves is complicated by the presence of three biochemical subtypes: NADP-ME, NAD-ME, and phosphoenolpyruvate carboxykinase (PEP-CK) [56]. C4 plants were classified into one of these subtypes based on their dominant C4 acid decarboxylation enzyme. Each of the C4 subtypes is characterized by specialized leaf anatomy, biochemistry, and physiology [50]. In the NAD-ME subtype, N requirement is greater relative to the NADP-ME subtype because the NAD-ME pathway involves more enzymatic steps and amino acids [48]. Moreover, the BS chloroplasts of NADP-ME grasses have a low amount of photosystem II (PSII) [45], which reduces the thylakoid N cost of leaves. Soluble protein represents a smaller fraction of leaf N in the NADP-ME subtype (41%) compared to the NAD-ME (53%) leaves, of which Rubisco accounts for one-seventh. The majority (65%) of leaf N and chlorophyll was found in the mesophyll cells of NADP-ME and BS of NAD-ME leaves. The mesophyll–BS distribution of functional thylakoid complexes (PSI and PSII, cytochrome b6f) varied among species. The high NUE of NADP-ME relative to NAD-ME grasses was achieved with less leaf N, soluble protein, and Rubisco having a faster *k*cat [57]. In C4 plants, Rubisco does not appear to limit photosynthesis at elevated temperatures because its in vitro capacity exceeds the net photosynthesis rate [58]. A higher *k*cat of Rubisco from C4 than C3 species is advantageous because less enzyme is required for photosynthesis. Because Rubisco in C4 plants operates in the very high CO_2_ concentration, the advantages of higher *k*cat are substantial [59]. For maize, it was reported that no correlation can be observed between Rubisco content and biomass, with respect to varying N levels, when the plants were grown at 25 °C (day temperature). Despite the reduction in photosynthetic enzymes, plants accumulate C in the form of starch under low-N conditions. This can be explained by the reduced demand of carbon for the assimilation of amino acids [60]. The increased biosynthesis of storage carbohydrates is associated with a decrease in sink strength in other parts of the plants, related to growth retardation of the shoot in nutrient stress [60,61]. A C3 species with high specific leaf nitrogen (SLN; the amount of N per unit of leaf area) usually has a high PNUE. The importance of PNUE in determining the NUE of a species to achieve optimal growth raises the question: what factor(s) causes species with high SLN to have higher PNUE at a given irradiance? This can be answered by the following: (1) there may be a difference between species in the fraction of light absorbed by the leaf cells; (2) plants may have similar CO_2_ response curves of photosynthesis, but they may operate at a different intercellular CO_2_ partial pressure; (3) the partial pressure of CO_2_ may be different at the carboxylation sites within the chloroplasts, or the CO_2_ assimilation rate per unit of photosynthetic N may be different because the plants partitioned N differently between light-harvesting complexes (LHCs), electron transport components, and CO_2_ fixation enzymes; and (4) there may be variations in the activation state or specific activity of Rubisco. In addition, there could be differences in the rate of respiration in the presence of light between plant species. Changes in dark respiration induced by light may be more complex in C4 plants because the variations in light penetration through the leaf blade may also influence the energy-demanding processes. It was shown that exposure of C3 leaves to light of different wavelengths results in differences in gradients in PSII yield and assimilation within the leaf [62]. The concentric anatomy of C4 leaves allows light to reach mesophyll cells before the deeper located BS cells [63] and to modify the light-harvesting process. In a previous study [64], we showed that the response of C4 plants to light is species-dependent because chloroplasts are differently stimulated, probably due to differences in light penetration across the leaves and also due to the redox status of the chloroplasts which influences C and N metabolism.

Stable energy supply is important for the regulation of metabolic processes in both mesophyll and BS cells. We found that the values of maximal PSII quantum yield (Fv/Fm) were similar in maize plants grown in different light intensities [14]. This is probably a photoprotective strategy for better utilization of the absorbed light at various light intensities. We also suggested that other additional mechanisms, instead of the xanthophyll cycle, may contribute to the dissipation of energy as heat in C4 plants. However, it is necessary to further characterize the roles of different xanthophylls in C4 plants, and the effect of N is still unknown. Especially in maize, in which CO_2_ concentration is high in BS chloroplasts, utilization of N is different than in mesophyll cells. This may be due to the differences in the content of N per unit of leaf area and variation in the amount of light required to saturate photosynthesis. It is still unclear whether N can induce changes in the organization of thylakoid complexes, and the impact of changes on their function and rate of photosynthesis needs to be investigated.

## 3. Thylakoid Nitrogen Costs

In maize, a gradient in the photosynthetic properties of chloroplasts occurs across the leaf, which is associated with leaf structure and biochemistry and is due to the formation of a leaf gradient of the light environment [63]. The changes induced by light in both types of chloroplasts are important in regulating the flexibility of the chloroplast membrane and thus its function. In maize, mesophyll chloroplasts are granal at all stages of development, whereas BS chloroplasts are fully agranal. Our previous study [45,65] demonstrated that the BS thylakoid membranes differ significantly from the stroma lamellae of C3 plants. The content of PSII is low in the agranal BS membranes, but PSII is a functional dimer with LHCII antennas and dominates the PSI complex. We also showed in a study [66] that LHCII antennas in BS chloroplasts are connected to PSI independent of light conditions, forming PSI–LHCI–LHCII supercomplex, and hence they may optimize cyclic electron flow and ATP production. Furthermore, mesophyll and BS chloroplasts use different mechanisms for adjusting and optimizing their functions, depending on the optimal or adverse irradiance conditions prevailing during growth, and these mechanisms are associated with different levels of light penetration across the leaf [14]. The intra leaf light gradient may increase the photosynthesis efficiency of C4 leaves with respect to light intensity and nitrogen utilization because the photosynthesis-related characteristics of chloroplasts vary across leaves. The intra leaf gradient of the photosynthetic properties of chloroplasts results in differences in the photosynthetic light response of both types of chloroplasts when the leaves are irradiated with lights of different intensities and quality. Terashima and Evans [67] showed that in spinach the cross-sectional area of chloroplasts was larger in leaves grown with higher nitrate concentrations and/or under lower irradiances. A larger chloroplast area results in a longer path for CO_2_ transport in the liquid phase, which may serve to lower the partial pressure of CO_2_ at the site of carboxylation. Therefore, it was observed that the ratio of in vitro activities of Rubisco was higher. For maize, adaptive strategies are not well documented, although the direct effect of the level of nitrogen on growth *per se* has been studied extensively.

To construct a leaf that can assimilate a large amount of carbon at high irradiances, a large investment of nitrogen is required. However, under LL conditions, a large N investment in the leaf to raise the rate of photosynthesis would not yield an appropriate return. Since the availability of nitrogen is often limiting in nature and much energy is required for the assimilation of nitrate into protein, it is essential for plants to use nitrogen efficiently. An investment of nitrogen or protein in a leaf “appropriate” to its environment must therefore be of adaptive significance. This idea is supported by strong correlations observed between the nitrogen content of leaves and growth irradiance [68]. As photosynthetic complexes are the major nitrogenous components in chloroplasts [34], the nitrogen cost of chlorophyll–protein (Chl–protein) and protein complexes should be studied in order to understand the role of nitrogen in photosynthetic production. It is well known that the chlorophyll a/b ratio increases with an increase in growth irradiance. At higher growth irradiances, a large amount of PSII core is required to realize a high rate of photosynthesis because the PSII core contains several electron carriers. By contrast, at lower irradiances, the relative amount of LHCII antenna increases since most chlorophyll b is associated with LHCII and the relative increase in LHCII among Chl–protein complexes in LL leaves causes a decrease in the chlorophyll a/b ratio [69]. It was shown that the nitrogen cost of Chl–protein complexes per leaf chlorophyll in typical leaves of LL-grown plants is 5–20% lower compared to HL-grown plants of the same species. Thus, the difference in the cost of light harvesting is significant, and the changes in the organization of Chl–protein complexes are of economic importance. However, it is unclear whether such drastic changes in the level of the PSI core relative to the LHCI level occur in response to changes in growth irradiance [70]. Nitrogen partitioned into Chl–protein complexes represents about 20% of photosynthetic nitrogen at a light intensity of 2000 μmol photons m^−2^ s^−1^, while it represents 50–60% at 125 μmol photons m^−2^ s^−1^. Thus, under LL conditions, Chl–protein complexes are the most nitrogeneous components in the leaf. This suggests that preferential investment of nitrogen in the components influencing the rate of photosynthesis the most important factor contributing to changes in the organization of photosynthetic complexes with changes in growth irradiance. Makino et al. [39] compared the allocation of nitrogen to photosynthetic components between a C4 plant (*Z. mays*) and a C3 plant (*Oryza sativa*) (Table 1).

It was found that the leaves of *Z. mays* allocated more N into the thylakoid components and showed greater rates of photosynthesis per unit of leaf N than *O. sativa*, even when CO_2_ was at the saturating level. The authors showed that N partitioning into insoluble fraction of thylakoid proteins was lower in rice (37%) than in maize (53%).

The total amount of thylakoid complexes accounted for 34% and 24% of leaf N in maize and rice, respectively. By contrast, the N partitioning into soluble protein fraction in a leaf was significantly lower in maize (33%) than in rice (50%). This was due to the presence of a smaller amount of Rubisco in maize (8.5%) than in rice (27%). The N costs of PEPC and PPDK were estimated at only 2.8% and 1.5%, respectively. The thylakoid membrane proteins associated with light harvesting, electron transport, and photophosphorylation may contribute to a higher capacity of RuBP regeneration. The number of thylakoid complexes varies in response to growth light intensity which also changes the rate of electron transport per unit of chlorophyll. Thylakoid nitrogen can be divided between two pools: light capture and bioenergetics. Nitrogen associated with these complexes can be estimated by determining the distribution of chlorophyll between them. Adding these fractions together, the average nitrogen cost for light capture was calculated at 37.3 mol N mol^−1^Chl [71]. The second thylakoid nitrogen pool is associated with photosynthetic electron transport and ATP synthesis. The cytochrome b6f and ATP synthase complexes abundance, depending on the growth light intensity, are correlated with the rate of electron transport [31]. Mu et al. [72] showed that plants invest more N to the bioenergetics pool to support electron transport and photophosphorylation, preferentially reduce PEPC, and use PPDK, Rubisco, and light-harvesting proteins, respectively, in response to low N stress. The plant growing in high light may have about 55 mol N mol^−1^Chl in chloroplast thylakoid membranes. A greater investment of nitrogen in the thylakoid components, as well as in CCM, supports higher PNUE in C4 plants.

In the case of mature leaves of C3 plants, the average distribution of N within the cell is as follows: chloroplast—75%, mitochondria—5%, peroxisomes—2.5%, cytosol and nucleus—7.5%, and cell walls—10% [63]. More attention should be paid to the changes in the light environment of the maize leaves during growth because irradiance received by a leaf more or less decreases with plant growth (shading of older leaves). Thus, in consequence, nitrogen availability and organization of the photosynthetic apparatus are different.

## 4. Nitrogen and Distribution of Organic Acids between Mesophyll and BS Cells in Different Light Environments

Organic acids are of fundamental importance in all plant species as they are involved in a number of metabolic pathways. These compounds play a major role in the C4 photosynthetic pathway as intermediates linking CO_2_ uptake and fixation. It is known that, in maize, not only the “classical” NADP-ME pathway involving decarboxylation of malate occurs in BS chloroplasts, producing NADPH, CO_2_, and pyruvate, but also aspartate (Asp) may be transported from mesophyll to BS cells (no NADPH is moved and extra ATP is required), resulting in different energy and nitrogen scenarios in both types of cells. Therefore, the presence of alternative decarboxylation pathways within maize allows the use of CCM under certain growth conditions [73]. It was proposed that the transport of malate and aspartate to BS cells plays an important role in adjusting energy and reducing equivalents to facilitate efficient photosynthesis in maize plants in changing environments [74]. Sharwood et al. [75] found that light availability differentially affects the production of organic acid in maize leaves, although PSII activity in BS, chloroplast structure of mesophyll and BS cells, and energy partitioning between these two cell types were not investigated. Synthesis of aspartate does compete with the production of malate and involves several regulatory steps. Aspartate aminotransferase is a link between nitrogen and carbon pathways, if aspartate is used to transport carbon to the bundle sheath cells. Thus, in maize, the alternative decarboxylation pathways may act as capacitors of ATP and reduce the equivalents needed for maintaining a high rate of photosynthesis in fluctuating light environments. Because in maize the subsets of metabolism are divided between the two cell types [10,76], there is also an expected change in the N pool. We found (data not published) that in LL intensity, the level of malate in mesophyll cells is about two times lower than in HL, whereas in BS cells the level is very similar in both light conditions. By contrast, the content of aspartate was two times higher in LL than in HL and was similar in both types of cells. This suggests that LL conditions stimulate the transport of aspartate and may increase the content of grana in BS chloroplasts. Thus, the amount of PSII and NADPH synthesis increases, and nitrogen is saved due to the lower amount of Rubisco, allowing for greater N investment in the thylakoid components.

## 5. Nitrogen Investment in the PEPC and PPDK Enzymes and Rubisco

C4 leaves change their photosynthetic characteristics in response to growth light conditions. It was observed that maize plants (NADP-ME C4 subtype) grown in HL conditions developed thicker leaves with higher maximum net photosynthesis rates per unit of leaf area compared to plants grown in LL [41]. The responses of C4 plants appear to differ depending on the decarboxylation subtype, type of plants (monocots or dicots), and even the species. For C3 leaves, models for photosynthesis with respect to nitrogen use were developed to explain the acclimation mechanisms of photosynthesis in response to light conditions during growth [70]. These models are based on theoretical predictions of optimum N investment into photosynthetic components under various levels of growth irradiance and nitrogen. According to these models, for different light environments and nitrogen contents of leaves, the amount of nitrogen allocated to Calvin cycle enzymes and electron carriers increases with increasing irradiance, while that allocated to Chl–protein complexes increases with decreasing irradiance. For the Chl–protein complex of PSII, the amount of LHCII relative to that of the core complex increases with decreasing irradiance. At any irradiance, N partitioning into Rubisco increases with an increase in the nitrogen content of leaves. The same approach can be applied to C4 plants. Sugiyama et al. [51] demonstrated that the leaves of *Z. mays* plants grown in HL conditions showed greater N allocation to the C4 enzymes: PEPC and PPDK. On the other hand, the proportion of Rubisco allocated to the soluble protein fraction did not vary depending on growth light—in contrast to C3 plants [77]. C4 leaves should invest their N into the C4 enzymes that are involved in the CO_2_-concentrating cycle [43]. Thus, maintaining the balance between C3 and C4 cycles is important for carrying out C4 photosynthesis efficiently, and N investment into Rubisco and C4 enzymes may be coordinately regulated. In maize plants, the allocation of nitrogen to Rubisco was almost constant irrespective of the leaf N, whereas allocation to PEPC increased with the increase of leaf N [39,59]. The difference in the N allocation pattern could be due to the difference in the regulation of photosynthetic gene expression [77,78]. NUE of plants is highly dependent on the interaction of environmental and genetic variations and results in adaptive phenotypes. Transcriptome analysis of maize leaves revealed differential nitrogen responses between genotypes and implicated a crucial role for transcription factors in driving genotype-by-nitrogen interactions at the level of gene expression [79]. The rate of photosynthesis is determined by the Rubisco content, and it should be noted that the ratio of photosynthesis rate to the Rubisco content decreases with the increase in enzyme content. This may indicate that the efficiency of Rubisco activity might be reduced in high-N leaves. In LL leaves, N investment into Rubisco appears to serve no purpose because these leaves do not receive HL.

It is known that the levels of the enzymes involved in the CO_2_-trapping system of C4 photosynthesis in mesophyll cells may be of more significance than that of Rubisco because PEPC and PPDK account for 14% and 8%, respectively, of total soluble proteins in maize plants grown under full sunlight [51], and must be directly proportional to maize biomass than the amount of Rubisco, at least under the near-optimal growth condition. The increase level of Rubisco (and concomitant decrease in the proportion of soluble protein allocated to PEPC and PPDK) at a lower N supply may indicate that the synthesis of Rubisco is a higher priority than other leaf soluble proteins when N is limiting, thereby compensating the lower capacity of CO_2_-uptake due to the insufficient content of these enzymes. There is no data on the N cost of other C4 enzymes. Thus, it is not known whether a lower amount of Rubisco is balancing by the N cost for total C4 cycle enzymes. Increase in N would lead to the rapid formation of PEPC and PPDK and eventually result in the increase in biomass. Among the two enzymes, the level of PEPC may have more influence on biomass yield in maize as is seen from its greater increase in proportion to soluble protein with N supply compared to PPDK under the optimal growth condition. Regardless of the growth condition, the decrease in soluble protein proportion allocated to Rubisco (about 10–15%) does not fully account for the increase in the proportions allocated to PEPC and PPDK [59]. This decrease may be recovered in other proteins which presumably limit the biomass formation of plants. Patel and Berry [80] showed that the translational control of Rubisco gene expression occurs during early leaf development, while control of mRNA stability mediates the accumulation of transcripts in mature leaves. The lack of correlation between the initial accumulation of mRNAs and Rubisco protein is explained as posttranscriptional regulation of gene expression by light in response to photosynthetic activity. The role of nitrogen supply in the regulation of gene expression is unknown. It was shown that light activates certain enzymes, for example, PEPC, NADP-malate dehydrogenase (NADP-MDH), and PPDK [81], localized in mesophyll cells. NADP-MDH is activated by the redox state, whereas PPDK and PEPC are activated by the phosphorylation/dephosphorylation of proteins. The mechanism of light activation may be controlled by the ATP/ADP ratio and light level [82]. The close relationships between maize biomass and the levels of PEPC and PPDK indicate that these enzymes might be useful targets for improving productivity by increasing NUE in maize plants.

## 6. Nitrogen Level and Light Intensity Affect the Amount and Activity of Nitrate Reductase (NR) and Nitrite Reductase (NiR)

Inorganic nitrogen concentrations in soil solutions vary among different soils and as a result of seasonal changes. Both NO_3_^−^ and NH_4_^+^ commonly serve as nitrogen sources for plant growth, and their concentrations in agricultural soils range from three to four orders of magnitude [83]. Plants have evolved numerous mechanisms for optimizing nitrogen acquisition. The physiological adaptations of plants include the “upregulation” of nitrogen uptake under conditions of N limitation and restriction of nitrogen uptake under conditions of N excess. Nitrate reduction is a highly regulated process [84]. In illuminated leaves, NO_3_^−^ assimilation is carried out with the use of chloroplast reductants and photosynthate to provide carbon skeletons for amino acids, and as a consequence, interaction occurs between C and N assimilation at the metabolic and energy level. The effect of nitrogen form (nitrate versus ammonium) on plant growth and photosynthesis has already been investigated [85]. Many plant species exhibit growth depressions when nitrogen is supplied in the form of ammonium, as ammonium nutrition has a negative effect on leaf area, relative growth rate, and DM yield [86]. The response of plants to ammonium is more pronounced under HL conditions because of photoinhibition. However, no differences in DM yield were found for different nitrogen forms under LL intensity. These results suggest that some of the adverse effects of ammonium nutrition on plant growth are related to photosynthesis. The fundamental differences in energy requirements for N assimilation between plants supplied with NO_3_^−^ and NH_4_^+^ should be related to leaf carbohydrate metabolism and ATP/NADPH balance, which are expected to affect the net CO_2_ assimilation. However, this phenomenon is poorly understood in the case of C4 plants. NR is a key enzyme that adjusts the processes of N assimilation and metabolism and is sensitive to changes in environmental conditions [87].

**Figure 3:**
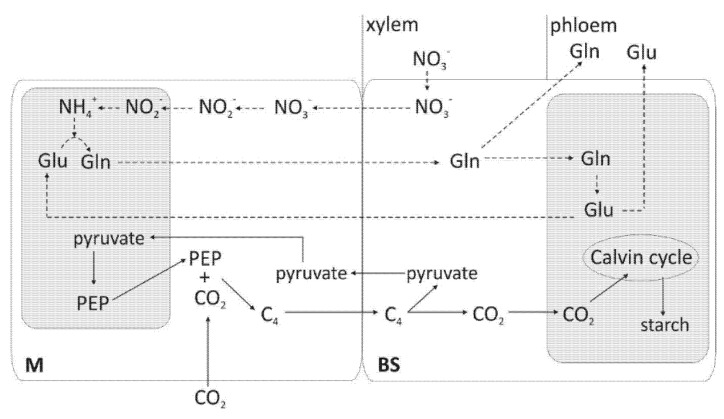
Schematic representation of the distribution of major steps in assimilation of carbon and nitrogen between mesophyll (M) and bundle sheath (BS) cells of maize. Dark-shaded rectangles represent chloroplasts; transport steps of C compounds are marked by solid arrows; dashed lines indicate the transport of N compounds. Gln: glutamine; Glu: glutamate. Modified by Kopriva and Kopriva [88].

The cell-specific distribution of assimilated N in C4 plants has been well established (Figure 3) [10]; however, many questions remain open. 

NR and NiR are restricted to the mesophyll cells and glutamate dehydrogenase to the BS cells of C4 plants, whereas glutamine synthetase (GS) and glutamate synthase (GOGAT) are active in both tissues. Limitation of NR to mesophyll cells is essential to prevent nitrite accumulation and toxicity in BS cells. Plants obtain ammonium from inorganic nitrogen, either by the reduction of nitrate or by direct uptake of ammonium from soil or through symbiotic associations with diazotrophic rhizobia in root nodules [89]. The secondary source is derived from organic compounds produced through processes such as photorespiration (inhibited in maize by high CO_2_ content). Upon nitrate transport through the vascular system into the BS and mesophyll cells, nitrate is reduced to nitrite by NR in the cytosol [29]. This nitrite is then imported into chloroplasts by nitrite transporters [90], followed by further reduction into ammonia by ferredoxin-nitrite reductase (Fd-NiR). Subsequently, ammonia is incorporated into glutamate in the GS/GOGAT pathway and redistributed to other amino acids through aspartate transaminase.

The primary nitrogen assimilation takes place in mesophyll cells, with Fd-NiR and ferredoxin-dependent glutamate synthase (Fd-GOGAT) predominantly localized to these cells, while GS activity occurs in both M and BS cells [71]. Fd-GOGAT was found [91] to be present in equal abundance in BS and mesophyll cells and participate in primary (in mesophyll) and secondary (in BS from photorespiration) nitrogen assimilation. NADH-GOGAT is strongly BS enriched (BS/mesophyll = 5.33), but its overall abundance is 100-fold lower than Fd-GOGAT. Accumulation of NADH-GOGAT in BS chloroplasts reflects metabolic adaptation to the environment of these chloroplasts. Inorganic nitrogen assimilation is strongly correlated with photosynthesis, and its distribution between BS and mesophyll cells must depend on the availability of reducing equivalents needed for Fd-NiR activity. The posttranslational regulation of NR activity (by dephosphorylation) illustrates that plants control N metabolism in relation to the rate of photosynthesis and environmental conditions for their optimal growth. Light plays a fundamental role in the regulation of glutamine synthetase [92]. Because sucrose enhances enzyme expression, it increases photosynthesis which in turn increases the expression of enzymes. This might be an evolutionary advantage of the cell-specific distribution of N assimilation in C4 plants. Since nitrate assimilation requires electrons to reduce NO_3_^−^ to NH_4_^+,^ the supply of different N forms can change the use of electrons in C and N assimilation. A range of environmental factors influence the expression of genes associated with N metabolism as well as the levels and activity of corresponding enzymes. NR expression and activity are controlled by light, temperature, pH, CO_2_, O_2_, water potential, and N source [40]. Leaf NR undergoes reversible phosphorylation in response to light/dark transitions, which leads to its inactivation. The low-activity, phosphorylated NR from darkened leaves is activated during purification. Transcriptome and metabolome profiling in maize seedlings [27] showed that low-N conditions reduced the level of phosphatases and contributed to improved binding of phosphate in organic compounds. Rogowski et al. [66] found that adjustment of light absorption and distribution between both photosystems in mesophyll and BS chloroplasts of maize is related to the organization of supercomplexes, according to the phosphorylation level. The level of LHCII phosphorylation was almost identical under different light intensities in BS thylakoids, whereas it decreased in mesophyll cells with the increase in light intensity. This suggests that mesophyll and BS chloroplasts use different mechanisms for the adjustment and optimization of their functions, depending on the irradiance conditions during growth. Thus, it seems that phosphorylation in different domains of thylakoid membranes plays a significant role in the light acclimation of photosynthesis.

The rising concentrations of atmospheric CO_2_ changes the C:N balance of plant tissues, reduces photorespiration, but plants have lower nitrogen concentrations [93]. This decreases the protein content of grains, which may have dietary implications in the future [94,95].

Differences in the total amount of leaf nitrogen and in the organization of photosynthetic components reported for plants from different environments would, therefore, be of adaptive significance because these can contribute to the realization of efficient photosynthesis. Although such differences show a similar trend in most higher plants, the range of environments to which the plants can adjust their photosynthetic apparatus appears to differ depending on the type of metabolism and species. These differences may result from the specialization of plants to particular ecological niches. To achieve sustainable agricultural production, it is necessary to grow crops that can efficiently uptake the nutrients from the soil and, therefore, require less fertilizer.

## 7. Main Conclusions and Perspectives

Photosynthesis and leaf nitrogen content of maize plants are affected by the availability of light during growth. There is a direct relationship between nitrogen content in mesophyll and bundle sheath cells and photosynthetic rate, modulated in response to environmental conditions to maximize net carbon gain.

Nitrogen metabolism in plants is controlled by several processes which are regulated at the molecular and genetic level. New technologies are providing opportunities to generate transgenic crops able to maintain high yields under changing environments. The identification of the key elements responding to N limitation in terms of light conditions will provide information on the effects of plant N status on grain formation. The response of leaf photosynthesis to irradiance is largely dependent on the leaf N content because photosynthetic proteins, including Rubisco and thylakoid protein complexes, represent a large proportion of total leaf N. The data concerning the impact of leaf N content on light-use efficiency are very limited, especially for C4 plants, where there is different C and N metabolism in mesophyll and bundle sheath cells. Plants grown in the field face heterogeneous conditions and are exposed to the simultaneous occurrence of different stresses. All presented data are from the laboratory conditions and refer to studies of interaction of light intensity and leaf N content in C4 plants, activity of main enzymes of C and N metabolism, abundance of thylakoid complexes, transport of metabolites. Such an approach will allow an understanding of those processes in a broader biochemical and molecular context. The regulation of leaf growth in relation to nitrogen supply, uptake, and N allocation between cell compartments in C4 plants in relation to irradiance are areas where research would improve the understanding of crop productivity.

## Figures and Tables

**Table 1:** Allocation of protein-N in young leaves of maize and rice [35].

	Protein-N (%)	
	Maize (C4)	Rice (C3)
Soluble protein	33	50
Insoluble protein	53	37
Rubisco	8.5	27
Thylakoid complexes (PSI, PSII, LHCII, cyt b_6_f, CF_1_/CF_0_)	34	24

## Data Availability

Not applicable.

## Abbreviations

APAR	photosynthetically active radiation
BS	bundle sheath
HL	high light
LL	low light
M	mesophyll
N	nitrogen
NUE	nitrogen use efficiency
PPDK	pyruvate phosphate dikinase
PEPC	phosphoenolpyruvate carboxylase
PEPCK	phosphoenolpyruvate carboxykinase
Pn	photosynthesis
PNUE	photosynthetic nitrogen-use efficiency
SLA	specific leaf area
SLN	specific leaf nitrogen
